# Marine-Lenhart Syndrome: Case Report, Diagnosis, and Management

**DOI:** 10.1155/2018/3268010

**Published:** 2018-10-24

**Authors:** Danielle Neuman, Russ Kuker, Francesco Vendrame

**Affiliations:** ^1^Division of Internal Medicine, Department of Medicine, University of Miami Miller School of Medicine/Jackson Memorial Hospital, Miami, FL, USA; ^2^Division of Nuclear Medicine, Department of Radiology, University of Miami Miller School of Medicine, Miami, FL, USA; ^3^Division of Endocrinology, Diabetes and Metabolism, Department of Medicine, University of Miami Miller School of Medicine, Miami, FL, USA

## Abstract

The coexistence of thyroid functioning nodules and Graves' disease is called Marine-Lenhart syndrome. This condition is estimated to occur in 0.8-2.7% of patients with Graves' disease with few cases reported in the literature. Criteria for the diagnosis are not well defined. Here, we present a case of hyperthyroidism characterized by the presence of stimulating TSH receptor antibodies and severe bilateral exophthalmos. A thyroid uptake and scan revealed an elevated 24-hour iodine-131 uptake and a discrete hot nodule in the upper pole of the right lobe which was also observed with a thyroid ultrasound. The patient was diagnosed with Marine-Lenhart syndrome complicated by thyroid eye disease and was treated with methimazole followed by thyroidectomy and orbital decompression. We review the existing literature and propose criteria for the diagnosis and treatment of this condition.

## 1. Introduction

Graves' disease, the most common cause of hyperthyroidism, is an autoimmune disorder caused by autoantibodies which activate the thyrotropin receptors of thyroid cells leading to an increased synthesis and release of thyroid hormones [1]. The coexistence of nodules can occur in up to 35% of patients and in 0.8-2.7% of cases these nodules are functioning adenomas, a combination that has been termed Marine-Lenhart syndrome [2–5]. Although several cases have been reported in the literature the criteria used for the definition of this condition are quite variable. Here we present the rare occurrence of Marine-Lenhart syndrome with thyroid eye disease. We provide a review of the existing literature and propose criteria for the diagnosis and management of this condition.

## 2. Case Presentation

**Figure 1:**
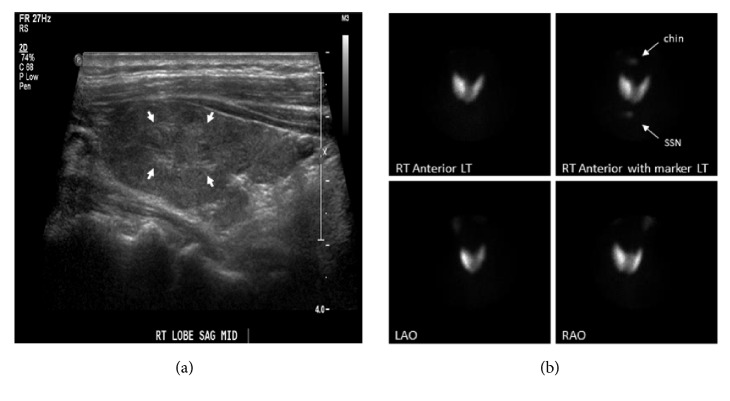
**Thyroid ultrasound and nuclear scan**. Grayscale sonographic image of the right thyroid lobe demonstrates a heterogeneous nodule with ill-defined borders (arrows) (a); technetium-99m pertechnetate thyroid scan showing increased uptake in both thyroid lobes with a more focal hot nodule in the superior to mid portion of the right lobe, corresponding to the nodule seen on ultrasound (b).

A 60-year-old Caucasian woman was referred to endocrinology division by her ophthalmologist because of abnormal thyroid tests. Her chief complaint for the past several weeks was bilateral eye pain and photophobia. She was symptomatic for occasional palpitations and mild shortness of breath. On physical examination, her blood pressure was elevated at 152/88 mmHg with a normal heart rate at 80 bpm. She presented with bilateral exophthalmos and an enlarged thyroid gland. She was on prednisone 20 mg twice daily as per the ophthalmologist's recommendation. Laboratory tests done two weeks prior revealed a suppressed TSH at 0.009 mIU/mL (0.270-4.200), an elevated FT4 at 4.59 ng/dL (0.93-1.70), and an elevated T3 at 231 ng/dL (80.0-200.0). The TSH Receptor Autoantibodies (TRAb) and Thyroid Stimulating Immunoglobulin (TSI) were both positive at 48.5 (<16.0 %) and 458 (<140 % baseline), respectively. New tests showed FT4 and T3 at 3.06 ng/dL and 242.1 ng/dL, respectively. The alanine aminotransaminase (ALT) level was elevated at 112 U/L (0-33). The patient was started on atenolol 25 mg daily and imaging studies were ordered. The thyroid ultrasound showed a mildly enlarged gland. In the right lobe, there was a heterogeneous solid nodule measuring 11 x 11 x 7 mm without calcifications (Figure 1(a)). A thyroid uptake and scan was performed and the 24-hour iodine-131 (I-131) uptake was calculated at 54%. On scintigraphy, the gland demonstrated increased uptake of technetium-99m pertechnetate and a discrete hot nodule in the upper pole of the right lobe corresponding to the nodule detected on ultrasound (Figure 1(b)). The patient was eventually started on methimazole 5 mg twice daily. Over the following weeks, ALT levels normalized and the dose of methimazole was increased to 30 mg daily. After receiving potassium iodine 1g/mL, 2 drops three times daily for one week, she underwent total thyroidectomy followed by bilateral orbital decompression one month later. Thyroid pathology was consistent with hyperplastic tissue and the diagnosis of Graves' disease.

## 3. Discussion

The coexistence of Graves' disease and functioning nodules is called Marine-Lenhart syndrome, but the criteria for its diagnosis are not well established. The name Marine-Lenhart syndrome was coined by David Charkes in 1972 when he described 10 patients with Graves' disease and functioning nodules characterized by the following characteristics: (1) when stimulated by TSH the nodules demonstrate increased radioiodine uptake which does not occur in the case of the hyper-functioning/autonomous nodules of Plummer's disease. The response is quantified 1.7-fold or greater; (2) the nodules demonstrate reduced radioiodine accumulation compared to the extranodular tissue and on scintigraphy appear “cool” which is not what is seen in patients with Plummer's disease; (3) these nodules are somehow resistant to radioiodine treatment and therefore require higher doses of I-131 compared to patients with Graves' disease; (4) after successful I-131 treatment these nodules demonstrate a relative increase in radioiodine uptake which does not occur in the case of Plummer's disease; (5) the pathology of these nodules consists of adenomas [3]. Serological data about autoimmunity, although reported, were not informative. Charkes estimated the prevalence of this disorder at approximately 2.7% based on a review of 375 cases of Graves' disease [3]. Another study reports a prevalence of 0.8% [5]. This condition was called Marine-Lenhart to honor the work done by David Marine and Carl H Lenhart at the beginning of the century when they published a study entitled “the pathological anatomy of exophthalmic goiter”[4]. The study included 8 cases where the thyroid pathology revealed the presence of adenomas with an iodine content inferior to the paranodular tissue, hence showing what Charkes observed years later. It remains unclear, however, if these patients really presented with a combination of both Graves' disease and functioning adenomas. It is also difficult to determine the extent to which the nodules described by Charkes contributed to the hyperthyroidism caused by Graves' disease. It is overall possible that these nodules are not different from the hyper-functioning/autonomous nodules of Plummer's disease. For example, a recent study shows that toxic nodules can also respond to TSH stimulation [23]. Possibly, the nodules described by Charkes are poorly functioning adenomas appearing “cool” because the iodine is taken up to a greater extent by the paranodular tissue [22].

Since the appearance of these first studies, others have reported similar observations. A PubMed search using the term “Marine-Lenhart” at the time of writing this report returned 33 articles and the most relevant are reported in Table 1. Both “cool,” although it is unclear if hyper-functioning, and “hot” nodules are described in patients with Graves' disease [9, 10, 12, 13, 15–21]. Cases of Marine-Lenhart with papillary thyroid carcinomas are also described [6–8, 14]. In order to reconcile the above discrepancies in the definition of the Marine-Lenhart syndrome, we propose the following criteria for the diagnosis and classification of patients with Graves' disease and functioning nodules: (1) thyroid function tests consistent with hyperthyroidism inclusive of serological testing for Graves' disease (TRAb/TSI); (2) increased radioiodine uptake and the presence of “cool” or “hot” nodules; thyroid nodularity should be supported by ultrasonography [24]; (3) thyroid nodule biopsy revealing a hyperplastic lesion or follicular adenoma, although, in the latter case, diagnostic surgery may be required to rule out a follicular carcinoma. Equally helpful is the final pathology in cases of thyroidectomy. According to the American Thyroid Association, cytological evaluation is generally not required for hyper-functioning nodules since they are rarely malignant [25]. However, for “hot” appearing nodules without proven hyper-function, the thyroid nodule biopsy can exclude the occurrence of discordant nodules which harbor malignancy [26]. An alternative is to perform a technetium-99m pertechnetate scan followed by an iodine-123 which can rule out the existence of discordant nodules. In the case we presented, the patient required a total thyroidectomy because of her severe ophthalmopathy but in the absence of those symptoms an iodine-123 scan could obviate the need for a thyroid biopsy if the nodule appeared hot on both scintigraphic studies. However, if the thyroid nodules biopsy is performed, this should be done based on the nodule size and the sonographic characteristics established by the American Thyroid Association [25]. We also suggest using the term “functioning” rather than autonomous nodule or Plummer's disease for consistency with the original definition given by Charkes [3]. Based on these criteria, it is then possible to distinguish a classic form of Marine-Lenhart, where nodules on scintigraphy present the “cool” appearance described by Charkes, from a variant form which includes all the cases characterized by “hot” appearing nodules. Subtypes of the latter include cases of functioning nodules with thyroid carcinomas and cases where Graves' disease and functioning nodules appear at different times on follow-up, for example, after methimazole or radioactive iodine treatment [11, 27]. Variant forms could also include cases characterized by the presence of functioning nodules able to suppress the uptake of the rest of gland in the presence of low levels of TRAb/TSI. In summary, the case presented in this report represents a variant form of Marine-Lenhart syndrome characterized by thyroid eye disease and abnormal liver function due to hyperthyroidism. The management of this condition relies on radioactive treatment or surgery with the latter being preferred in the case of malignancy, moderate to severe orbitopathy, large or symptomatic compressing goiters, and patient preference. In select patients such as those with severe hyperthyroidism or advanced age, thionamide therapy can be used to achieve euthyroidism before definitive treatment, noting that one of the major adverse effects of thionamide drugs, although not common, is hepatotoxicity. Accordingly, patients with abnormal liver function at baseline, such as the case we presented, should be monitored for signs of liver failure.

## Figures and Tables

**Table 1:** **Diagnosis of Marine-Lenhart syndrome in the literature:** most recent to oldest.

***Author*** **(*Ref*)**	**HT**	**TRAb/TSI**	**Thyroid ultrasound**	**Nodule on scan**	**Post-treatment scan**	**Pathology**
[6]	+	+	Yes	H	Yes^a^	PTC
	+	+	Yes	H	No	B
[7]	+	-	Yes	H	Yes^a^	PTC
[8]	+	+	Yes	H/C^b^	Yes^a^	PTC
[9]	SC	+	Yes	H^c^	Yes	N/A
[10]	+	-	Yes	H	No	N/A
[11]	+^d^	+^d^	Yes^d^	H^d^	Yes	N/A
[12]	+	N/A	Yes	C	No	B
[13]	+	N/A	N/A	H	No	N/A
[14]	+	+	Yes	H	No	PTC
[15]	+	N/A	Yes	C	No	B
[16]	+	N/A	N/A	C	No	N/A
[17]	+	-	Yes	H^c^	Yes	N/A
[18]	+	+	Yes	H	No	N/A
[19]	+	-	No	H	No	N/A
[20]	+	+	N/A	H	No	N/A
[21]	+	+	Yes	H	Yes	FTA
[22]	+	N/A	N/A	^ab^	Yes	FTA

Abbreviations: B = benign pathology; C = cold; FTA = follicular thyroid adenoma; H = hot; HT = hyperthyroidism; N/A = not available; SC = subclinical; PTC = papillary thyroid carcinoma; TRAb = TSH receptor antibody; TSI = thyroid stimulating immunoglobulin.

^a^whole body scan performed after treatment with radioactive iodine or surgery; ^b^focal uptake with centrally hypoactive foci before treatment; ^c^hot nodule detected post-treatment with radioactive iodine; ^d^patient was treated with radioactive iodine, achieved euthyroidism, and became negative for TRAb; a hot nodule was detected on follow-up on both ultrasound and nuclear scan; ^ab^cold nodule before treatment and hot nodule after treatment with radioactive iodine.
